# Erfolgreiche Behandlung einer palmoplantaren Pustulose mit Bimekizumab und topischer Photochemotherapie bei gleichzeitig bestehender Hidradenitis suppurativa

**DOI:** 10.1111/ddg.15986_g

**Published:** 2026-03-09

**Authors:** Neda Cramer, Johannes Mohr, Michael P. Schön, Rotraut Mössner

**Affiliations:** ^1^ Klinik für Dermatologie Venerologie and Allergologie Universitätsmedizin Göttingen Göttingen Germany

**Keywords:** Bimekizumab, Palmoplantare Pustulose, Psoriasis, Bimekizumab, Palmoplantar pustulosis, Psoriasis

Sehr geehrte Herausgeber,

Die palmoplantare Pustulose (PPP) ist eine chronisch‐rezidivierende entzündliche Hauterkrankung, die durch sterile Pusteln, Erythem und Hyperkeratosen an Handflächen und/oder Fußsohlen gekennzeichnet ist. Sie verursacht Schmerzen und Juckreiz und kann die Lebensqualität beeinträchtigen.^1^, ^2^ Die Behandlung der PPP ist herausfordernd, und standardisierte Therapiempfehlungen fehlen. Bimekizumab, ein dualer Interleukin (IL)‐17A‐ und IL‐17F‐Inhibitor, ist zur Behandlung der Plaque‐Psoriasis, Psoriasis‐Arthritis, axialen Spondyloarthritis und Hidradenitis suppurativa (HS) zugelassen.^3^ Darüber hinaus hat sich Bimekizumab auch als vielversprechende therapeutische Option für die PPP erwiesen.^4^ Hier berichten wir über einen Fall einer Patientin mit schwerer PPP und HS, die erfolgreich mit einer Kombination aus Bimekizumab und topischer Photochemotherapie mit Psoralen plus UVA (PUVA) behandelt wurde.

Eine 62‐jährige europäische Patientin stellte sich mit HS und PPP vor. Die HS hatte sich erstmals vor 10 Jahre manifestiert und wurde bisher zweimal chirurgisch behandelt. Zudem zeigte die HS kein ausreichendes Ansprechen auf eine sechsmonatige systemische Therapie mit Clindamycin und Rifampicin (jeweils 600 mg/Tag). Klinisch präsentierte sich die HS inguinal und genital im Hurley‐Stadium II. An Handflächen und Fußsohlen bestanden zudem seit sechs Monaten – also bereits vor Einleitung einer systemischen Therapie der HS – juckende, scharf begrenzte erythematosquamöse Plaques und Pusteln (Abbildung 1a).

**ABBILDUNG 1:**
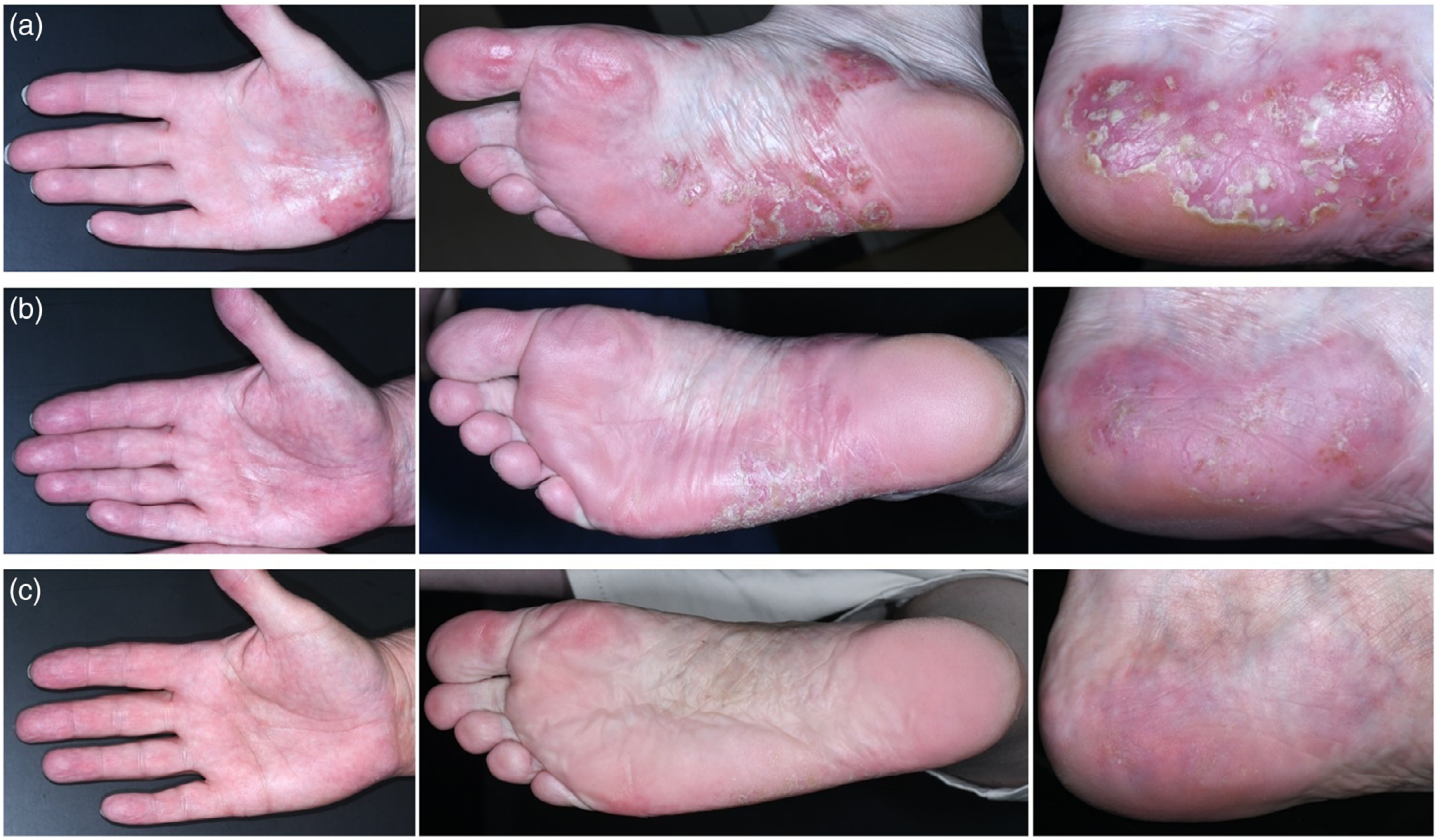
Klinische Bilder der rechten Handfläche, der rechten Fußsohle und der linken Ferse (a) vor sowie (b–c) nach Beginn der Therapie mit Bimekizumab in Kombination mit Phototherapie mit Psoralen plus UV‐A (b: nach einer Dosis Bimekizumab 320 mg 4 Wochen nach Therapiebeginn, c: nach insgesamt drei Dosen Bimekizumab 320 mg 13 Wochen nach Therapiebeginn mit Bimekizumab).

Es lagen weder eine Plaque‐Psoriasis noch Gelenkbeschwerden vor. Die Familienanamnese für Hauterkrankungen war unauffällig. Die Patientin war aktive Raucherin mit einer 15‐*Pack‐Years*‐Anamnese. Der BMI betrug 30,5. Die Komorbidität umfasste außerdem eine arterielle Hypertonie, ein Bandscheibenprolaps C7/8 sowie eine Hypothyreose, die mit Candesartan beziehungsweise L‐Thyroxin behandelt wurden. Die tägliche topische Therapie mit Mometason oder Clobetasol führte nicht zu ausreichender Besserung, und es traten weiterhin Schübe auf. Zur Behandlung der HS wurde eine Therapie mit Bimekizumab in einer Dosierung von 320 mg alle zwei Wochen über einen Zeitraum von 16 Wochen eingeleitet, gefolgt von einer Erhaltungsdosis alle vier Wochen. Zusätzlich beabsichtigte dieses Vorgehen, auch die PPP wirksam zu kontrollieren, gestützt auf vielversprechende, wenn auch bislang begrenzte Daten.^4^ Zum Zeitpunkt der Behandlung betrug die Krankheitsdauer der PPP 21 Monate. Aufgrund eines Missverständnisses wurde Bimekizumab jedoch im Abstand von vier Wochen und nicht – wie für HS zugelassen – alle zwei Wochen verabreicht.

Vor Therapiebeginn wurde der Juckreiz an Handflächen und Fußsohlen mit 10 von 10 Punkten auf der Numerischen Ratingskala (NRS) angegeben. Der *Palmoplantar Pustulosis Area and Severity Index* (PPPASI) betrug 11,2 (Abbildung 1a), der *Dermatology Life Quality Index* (DLQI) lag bei 19 und wies damit auf eine erhebliche Beeinträchtigung der Lebensqualität hin. Unter der Therapie mit Bimekizumab erfolgte eine topische PUVA‐Behandlung drei‐ bis viermal pro Woche über acht Wochen, anschließend zweimal pro Woche. 8‐Methoxypsoralen wurde 30 Minuten vor der Bestrahlung auf Handflächen und Fußsohlen appliziert; die PUVA‐Therapie wurde mit 0,50 Joule (J)/cm^2^ begonnen und wurde durch die niedergelassene Dermatologin der Patientin fortgeführt. Die Applikation von Mometason‐Creme wurde in den ersten drei Wochen schrittweise reduziert und anschließend beendet.

Nach vierwöchiger Therapie mit Bimekizumab zeigte sich eine deutliche Besserung der PPP mit einem Rückgang des PPPASI auf 5,5 (Abbildung 1b). Nach 13 Wochen sank der PPPASI weiter auf 0,9 (Abbildung 1c), der DLQI betrug 0. Als einziges unerwünschtes Ereignis trat nach 11 Behandlungswochen ein fieberhafter gastrointestinaler Infekt auf, der innerhalb einer Woche vollständig abklang. Die inguinalen HS‐Läsionen blieben während der gesamten Behandlung jedoch weiterhin ausgeprägt und entzündet. Bereits vor Therapiebeginn bestanden im Bereich der HS‐Läsionen erythematöse Papeln und Pusteln, die klinisch am ehesten mit einer Follikulitis vereinbar waren. Mykologische Untersuchungen waren negativ. Die topische Behandlung mit einem Kombinationspräparat aus Halometason und Triclosan besserte die Papeln und Pusteln.

Die therapeutischen Herausforderungen der PPP ergeben sich aus ihrem chronisch‐rezidivierenden Verlauf und der eingeschränkten Wirksamkeit konventioneller Therapien. IL‐17A‐Inhibitoren haben ein gewisses Potenzial in der Behandlung der PPP gezeigt. Während eine Phase‐III‐Studie mit dem IL‐17A‐Blocker Secukinumab den primären Endpunkt nicht erreichte, konnte mit Brodalumab, einem Inhibitor des IL‐17 Rezeptors A (IL17RA), in einer Phase‐III‐Studie eine signifikante Wirksamkeit nachgewiesen werden.^5^, ^6^, ^7^ Bimekizumab weist eine deutlich höhere Bindungsaffinität zu IL‐17A auf als Secukinumab.^8^ Zudem konnte gezeigt werden, dass sowohl IL‐17A als auch IL‐17F in PPP‐Läsionen erhöht exprimiert sind, was zur Rekrutierung von Neutrophilen und zu verstärkten lokalen Immunreaktionen beitragen könnte.^9^, ^10^ Bimekizumab könnte daher die inflammatorische Kaskade bei PPP wirksamer unterdrücken als Secukinumab. Die Kombination aus topischer PUVA und Bimekizumab adressiert in diesem Fall unterschiedliche pathogenetische Signalwege und könnte durch synergistische Effekte eine gesteigerte Wirksamkeit entfalten. Frühere Studien haben gezeigt, dass durch die Kombination von PUVA mit systemischen Wirkstoffen wie Acitretin oder Methotrexat verbesserte Behandlungsergebnisse erzielt werden können.^11^, ^12^ Für die Kombination von PUVA mit IL‐17‐Inhibitoren bei PPP liegen bislang keine Daten vor, die vorhandene Evidenz stützt jedoch die Plausibilität eines solchen Therapieansatzes.

Die Wirkung von Bimekizumab auf die HS bei unserer Patientin kann zu diesem Zeitpunkt nicht abschließend beurteilt werden, da während der Dosiseskalationsphase bis Woche 13 jede zweite Dosis nicht verabreicht wurde. Dadurch wurden insgesamt weniger Dosen appliziert (nur drei Dosen zu je 320 mg statt sechs). In Phase‐III‐Studien zur HS wurde der primäre Wirksamkeitsendpunkt in Woche 16 gemessen, wobei viele Patienten bereits zwischen Woche 4 und 12 eine klinisch relevante Verbesserung zeigten.^13^ Die Daten deuten zudem darauf hin, dass die therapeutische Antwort über diesen Endpunkt hinaus weiter zunimmt.^13^ Auf Basis dieser Evidenz planen wir, die Therapie fortzusetzen und die Reevaluation der Wirksamkeit frühestens in Woche 24 unter Verwendung der zugelassenen Dosierung vorzunehmen. Bei unzureichendem Ansprechen auf Bimekizumab könnte Adalimumab eine therapeutische Alternative darstellen, entsprechend seiner zugelassenen Indikation für HS, und damit das Potenzial bieten, sowohl HS als auch PPP zu behandeln.^14^, ^15^, ^16^

Bezüglich der PPP zeigt unser Fallbericht die ausgezeichnete Wirksamkeit von Bimekizumab in Kombination mit PUVA‐Therapie, verbunden mit einem raschen Wirkungseintritt. Bereits nach vier Wochen kam es zu einer 50%‐igen Besserung der Hautläsionen und des PPPASI, und nach 13 Wochen wurde nahezu eine komplette Abheilung erreicht. Bislang existieren keine veröffentlichten Daten zur Behandlung der PPP mit Bimekizumab, abgesehen von einer Fallserie.^4^ In dieser kürzlich publizierten, retrospektiven multizentrischen Fallserie mit 21 Patienten zeigte Bimekizumab eine deutliche Wirksamkeit bei Patienten mit PPP oder mit palmoplantaren Plaque‐Psoriasis‐Läsionen mit Pusteln. Bemerkenswert ist, dass 17 von 21 Patienten innerhalb von ein bis vier Monaten eine vollständige Remission erreichten.^4^

Zusammen mit unserem Fallbericht deuten diese Ergebnisse darauf hin, dass Bimekizumab in Kombination mit topischer PUVA‐Therapie ein vielversprechender neuer Ansatz zur Behandlung schwerer und therapieresistenter PPP sein könnte.

## INTERESSENKONFLIKT

J.M. war als Berater tätig und/oder erhielt Fördermittel und/oder nahm an klinischen Studien derbfolgenden Unternehmen teil: Abbvie, Allmirall, Biogen IDEC, Böhringer‐Ingelheim, Celgene, Janssen‐Cilag, Leo Pharma GmbH, Lilly, MSD SHARP & DOHME, Novartis Pharma, Pfizer und UCB.

R.M. war als Berater tätig und/oder erhielt Vortragshonorare und/oder Fördermittel und/oder nahm an klinischen Studien der folgenden Unternehmen teil: AbbVie, Amgen, Almirall, Biogen IDEC, Böhringer‐Ingelheim, Celgene, Janssen‐Cilag, Leo Pharma, Lilly, Moonlake, MSD SHARP & DOHME, Novartis Pharma, Pfizer und UCB.

M.P.S. war Berater und/oder erhielt Vortragshonorare und/oder Zuschüsse und/oder nahm an klinischen Studien folgender Unternehmen teil: AbbVie, Almirall, Biogen, Boehringer‐Ingelheim, BMS, Celltrion, Janssen‐Cilag, Leo, Lilly, Novartis, Scinai, UCB.

N.C. gibt keine Interessenkonflikte an.

## DANKSAGUNG

Open access Veröffentlichung ermöglicht und organisiert durch Projekt DEAL.
